# Supplementary material for: Sound-based assembly of three-dimensional cellularized and acellularized constructs
Source: Mater Today Bio. 2023 Aug 19;22:100775. doi: 10.1016/j.mtbio.2023.100775 (PMC10477805; doi:10.1016/j.mtbio.2023.100775)
Supplement: Multimedia component 1 [file mmc1.docx]

# Supplementary information

# Sound-based assembly of three-dimensional cellularized and acellularized constructs

*Riccardo Tognato^1^, Romedi Parolini^1^, Shahrbanoo Jahangir^1^, Junxuan Ma^1^, Sammy Florczak^2, 3^, R. Goeff Richards^1^, Riccardo Levato^2, 3^, Mauro Alini^1^, Tiziano Serra^1, 4, #^.*

Affiliation:

^1^ AO Research Institute Davos, Switzerland

^2^ Regenerative Medicine Center Utrecht and Department of Clinical Sciences, Faculty of Veterinary Medicine, Utrecht University, Utrecht, The Netherlands

^3^ Department of Orthopaedics, University Medical Center Utrecht, Utrecht University, Utrecht, The Netherlands

^4^ MERLN Institute for Technology-Inspired Regenerative Medicine, Maastricht University

^#^ Corresponding author: [Tiziano.Serra@aofoundation.org](mailto:Tiziano.Serra@aofoundation.org), Clavadelstrasse 8, 7270, Davos, Switzerland


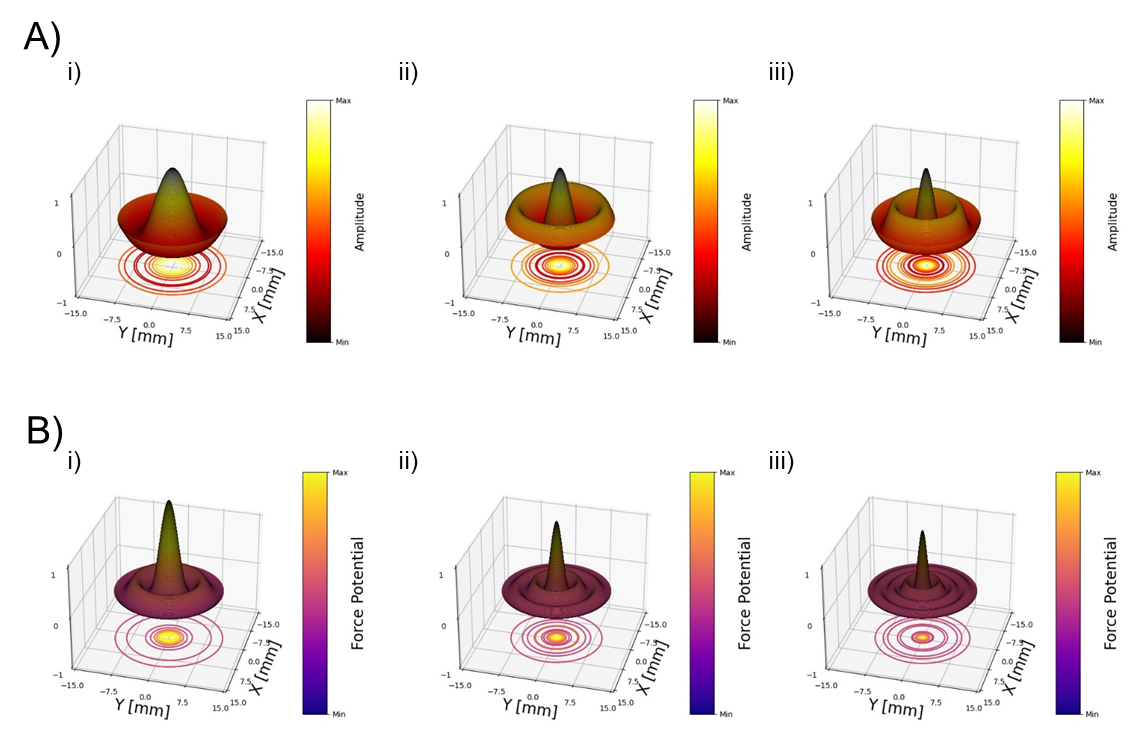


**Figure S1**. Numerical simulation surface displacements (A) and force potential (B) for one (i), two (ii) or three (iii) nodal circles. Simulations are based on the work proposed by Lei *et al.*[1] and Ren *et al.*[2]


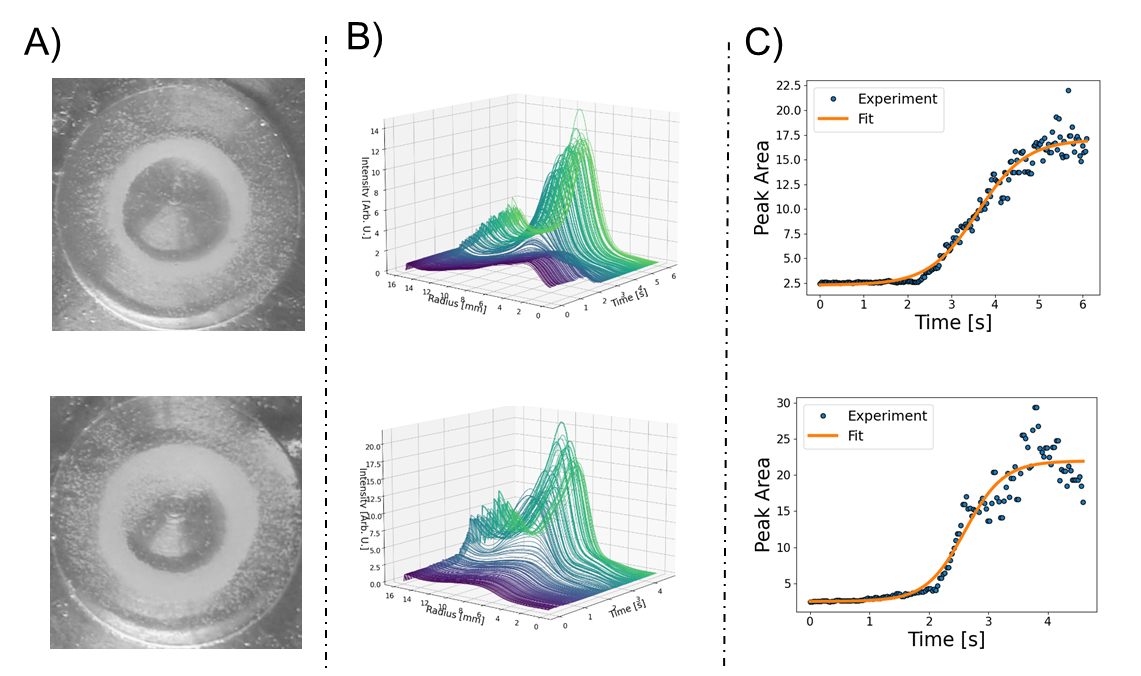


**Figure S2**. Repetition of the assembly kinetic of β-TCP particles in GelMA 5 % w/v. (A) Final structure, (B) radial profile analysis over time and (C) curve area as function of assembly time.


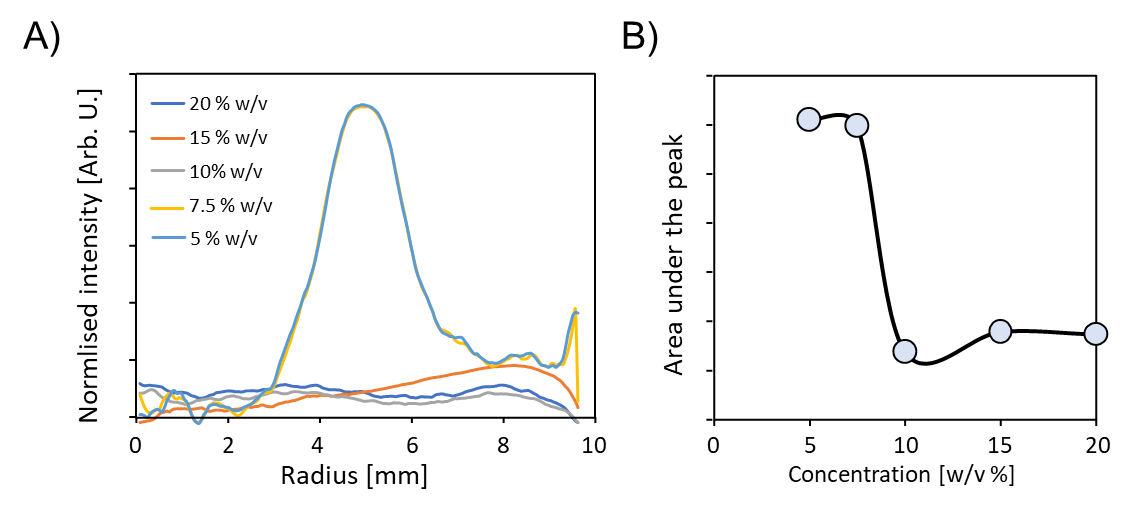


**Figure S3**. A) radial profile evaluation of sample patterned at increasing concentration of GelMA. B) Area under the peak of the profile shown in (A) as function of the prepolymer concentration.


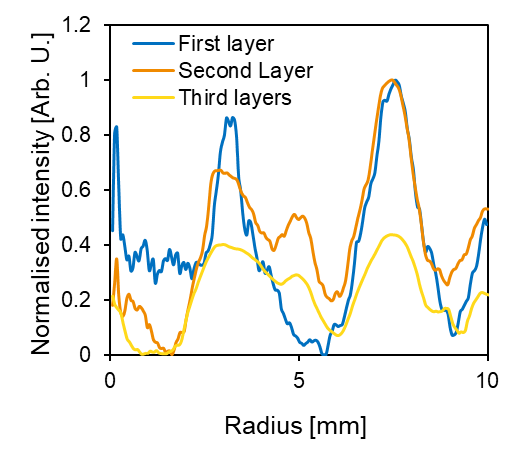


**Figure S4**. Radial profile analysis of a multilayered sample reported in Fig. 3-B.


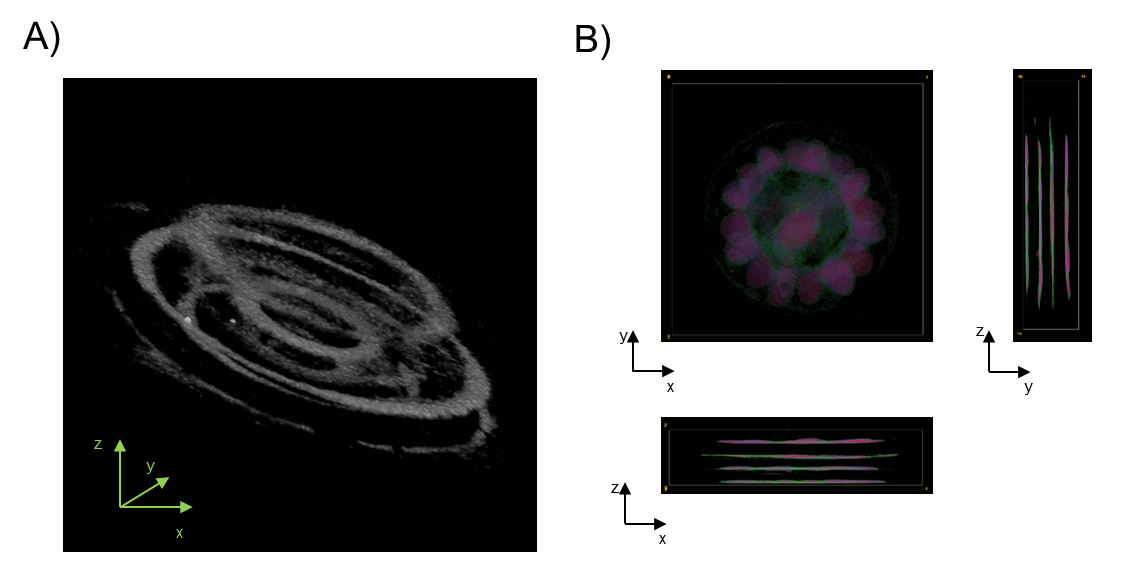


**Figure S5**. 3D reconstruction (A) and 3D projection (B) of the CT scan for the multilayered sample in the circular frame.


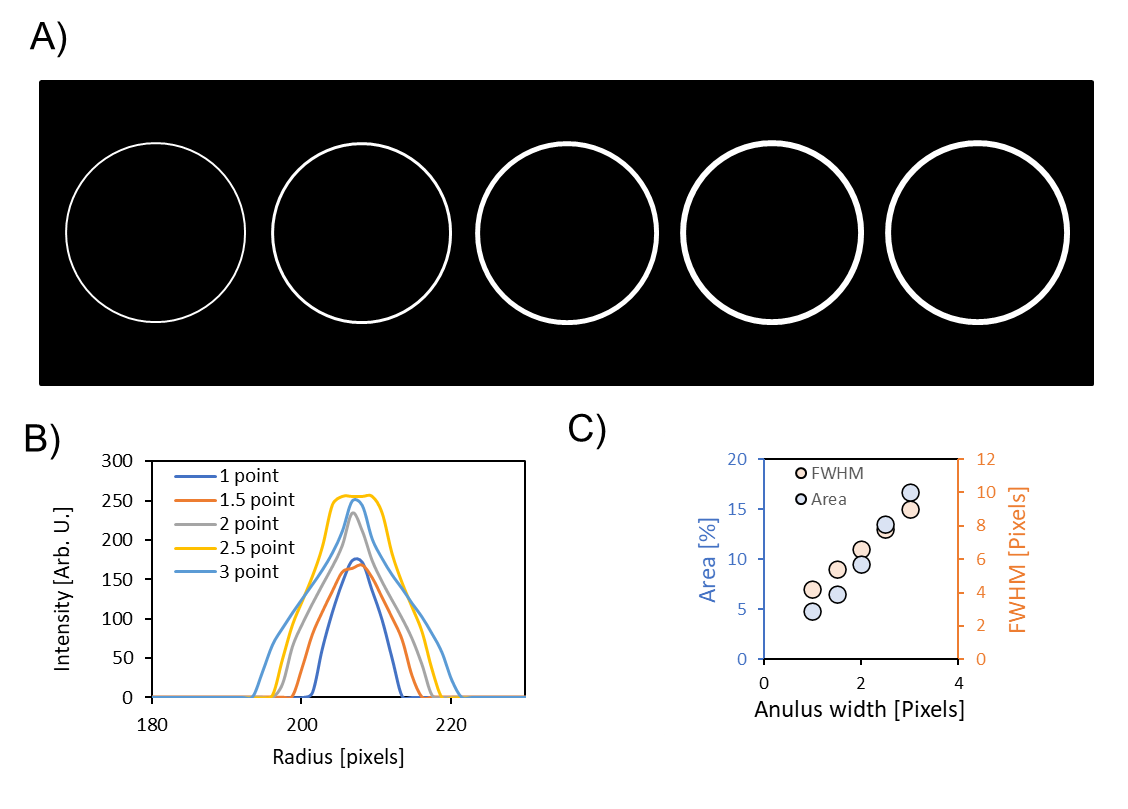


**Figure S6**. Simulated data for cell sprouting from the original annular region (A), radial profile (B) and quantification of the full width half maximum and area coverage (C).

RT-qPCR:

**Runx2**

| forward primer seq | 5'-AGC AAG GTT CAA CGA TCT GAG AT-3' |
| --- | --- |
| reverse primer seq | 5'-TTT GTG AAG ACG GTT ATG GTC AA-3' |
| probe seq | 5'-TGA AAC TCT TGC CTC GTC CAC TCC G-3' |

**ALP:** Assay on-demand

| **Assay ID** | Hs00758162_m1 |
| --- | --- |

**Osteocalcin**

| forward primer seq | 5'-AAG AGA CCC AGG CGC TAC CT-3' |
| --- | --- |
| reverse primer seq | 5'-AAC TCG TCA CAG TCC GGA TTG-3' |
| probe seq | 5'-ATG GCT GGG AGC CCC AGT CCC-3' |

**Colla1**

| forward primer seq | 5'-CCC TGG AAA GAA TGG AGA TGA T-3' |
| --- | --- |
| reverse primer seq | 5'-ACT GAA ACC TCT GTG TCC CTT CA-3' |
| probe seq | 5'-CGG GCA ATC CTC GAG CAC CCT -3' |

**Osteopontin**

| forward primer seq | 5'-CTC AGG CCA GTT GCA GCC-3' |
| --- | --- |
| reverse primer seq | 5'-CAA AAG CAA ATC ACT GCA ATT CTC-3' |
| probe seq | 5'-AAA CGC CCA AGG AAA ACT CAC TAC C-3' |

**Sp7**

| forward primer seq | 5'-CCT GCT TGA GGA GGA AGT TCA-3' |
| --- | --- |
| reverse primer seq | 5'-GGC TAG AGC CAC CAA ATT TGC-3' |
| probe seq | 5'-TCC CCT GGC CAT GCT GAC GG-3' |

**RPLP0**

| forward primer seq | 5'-TGG GCA AGA ACA CCA TGA TG-3' |
| --- | --- |
| reverse primer seq | 5'-CGG ATA TGA GGC AGC AGT TTC-3' |
| probe seq | 5'-AGG GCA CCT GGA AAA CAA CCC AGC-3' |

# Bibliography

[1] J. Lei, Formation of inverse Chladni patterns in liquids at microscale: roles of acoustic radiation and streaming-induced drag forces, Microfluid. Nanofluidics. 21 (2017). https://doi.org/10.1007/s10404-017-1888-5.

[2] T. Ren, P. Chen, L. Gu, M.G. Ogut, U. Demirci, Soft Ring-Shaped Cellu-Robots with Simultaneous Locomotion in Batches, Adv. Mater. 32 (2020). https://doi.org/10.1002/adma.201905713.
