# Supplementary figures and images for: Sound-based assembly of three-dimensional cellularized and acellularized constructs
Source: Mater Today Bio. 2023 Aug 19;22:100775. doi: 10.1016/j.mtbio.2023.100775 (PMC10477805; doi:10.1016/j.mtbio.2023.100775)

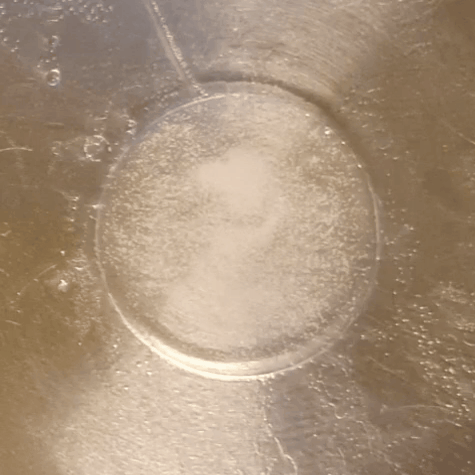

Supplement: Multimedia component 2 [file mmc2.gif]
